# CARF activates beta-catenin/TCF signaling in the hepatocellular carcinoma

**DOI:** 10.18632/oncotarget.13138

**Published:** 2016-11-05

**Authors:** Xin Fan, Xiaoyan Ma, Lei Cui, Shengchun Dang, Jianguo Qu, Jianxin Zhang, Xuqing Wang, Zhengfa Mao

**Affiliations:** ^1^ Department of General Surgery, Affiliated Hospital of Jiangsu University, Zhenjiang, Jiangsu Province, PR China; ^2^ Department of Gynecology and Obstetrics, Affiliated Hospital of Jiangsu University, Zhenjiang, Jiangsu Province, PR China

**Keywords:** CARF, HCC, beta-catenin/TCF signaling, Ras^V12^

## Abstract

Overactivation of Ras signaling is very common in the hepatocellular carcinoma (HCC) due to its constitutive active mutation, which makes it a big challenge to target Ras signaling. Therefore, identifying effectors downstream of Ras signaling would benefit the development of novel therapeutic strategies. In this study, it was found that the expression of CARF (collaborate of ARF) was induced by oncogenic Ras^V12^. The expression of CARF was up-regulated in both HCC mouse model (Alb-Cre; P53^f/f^; Loxp-Stop-Loxp-Ras^G12D^) and human HCC clinical samples. Overexpression of CARF promoted the growth and migration of HCC cells, while knocking down the expression of CARF inhibited the growth and migration of HCC cells. In the mechanism study, CARF was found to interact with beta-catenin, impaired the interaction between beta-catenin and ICAT, and activated beta-catenin/TCF signaling. Moreover, knocking down the expression of CARF inhibited the tumorigenesis in the HCC mouse model. Taken together, this study revealed the oncogenic functions of CARF in the tumorigenesis of HCC by activating beta-catenin/TCF signaling, and suggested CARF might be a therapeutic target in the treatment of HCC.

## INTRODUCTION

Hepatocellular carcinoma (HCC) is one of the most common malignancies in the world and its incidence is increasing yearly [1]. Although surgical operation and chemotherapy have played the crucial roles in the treatment of HCC, the survival of HCC patients is still very poor. Therefore, better understanding the molecular mechanisms driven HCC would benefit the clinical treatment.

Over-activation Ras signaling due to its constitutive mutation on the 12th code is frequently observed in HCC clinical samples [2]. Oncogenic Ras signaling promoted the growth, migration and malignant transformation of HCC cells. Moreover, recent studies have shown that oncogenic Ras signaling reprogrammed the metabolic profile of cancer cells [3], suggesting the pivotal roles of Ras in the progression of HCC. However, the development of small molecular inhibitors targeting Ras signaling is unsuccessful up to date. Identifying the effectors downstream of Ras would provide novel target.

Besides oncogenic Ras signaling, dys-regulation of beta-catenin/TCF signaling could also be found in most of HCC clinical samples [4]. Wnt ligand simulation or activation of growth factor receptor signaling leads to the accumulation of beta-catenin in the cytoplasm and subsequent nuclear localization [5]. In the nucleus, beta-catenin formed a complex with TCF4 and regulated the expression of multiple genes including Cyclin D1, Snail and so on [6]. Overactivation of beta-catenin/TCF signaling promoted the growth, migration and malignant transformation of HCC cells. ICG001 which blocked the interaction between TCF4 and beta-catenin inhibits beta-catenin/TCF signaling and showed anti-cancer effects on the HCC cells [7].

Cross-talk between oncogenic Ras signaling and beta-catenin/TCF signaling is very common in the tumorigenesis. Ras-activated Dsor1, the homolog of Raf, has been reported to promote the beta-catenin signaling in Drosophila development [8]. Consistently, Sorafenib, the inhibitor of Raf, inhibited beta-catenin/TCF signaling in the tumorigenesis of HCC [9], suggesting that beta-catenin/TCF signaling could be regulated by Ras. Further identifying the effectors mediating the cross-talk between beta-catenin/TCF signaling and Ras signaling would provide novel insight for the development of therapeutic drug.

CARF, collaborator of ARF (alternative reading frame), activates p53 functions by direct interacting and stabilizing of ARF as well as p53 proteins [10]. In addition, CARF inhibited the transcription of HDM2 (human homologue of mouse double minute 2), the antagonist of P53 [11]. Overexpression of CARF led to upregulation of p53 and triggered anti-proliferative signaling, its suppress-expression resulted in downregulation of p53 and elicited proproliferative signaling suggesting that CARF may regulate cell proliferation differently in cancer cells with variable p53 status [12]. In this study, we examined the expression and functions of CARF in HCC.

## RESULTS

### The expression of CARF was induced by the oncogenic Ras and necessary for the malignant transformation

**Figure 1:**
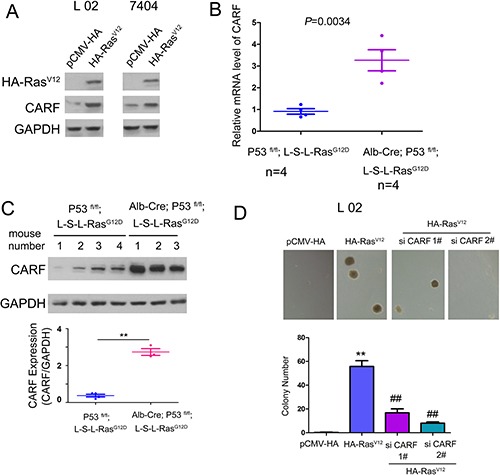
The expression of CARF was induced by oncogenic Ras (**A**) Ras^V12^ induced the expression of CARF. HA-tagged Ras^V12^ was over-expressed in 7404 and L02 cells, and the expression of CARF was examined using western blot. The experiments were performed three times. (**B**) Up-regulation of CARF mRNA in the HCC mouse model (Alb-Cre; P53^f/f^; Loxp-Stop-Loxp-Ras^G12D^) compared with the control mice. The 18S was used as the internal control. The expression level of CARF mRNA in control group was normalized to 1. (**C**) Up-regulation of CARF protein level in the HCC mouse model (Alb-Cre; P53^f/f^; Ras) compared with the control mice. The CARF protein was detected using western blot and quantified. (**D**) Knocking down the expression of CARF inhibited the malignant transformation driven by Ras^V12^ demonstrated by the colony formation assay on the soft agar. The experiments were performed in triplicates. **P < 0.01.

Constitutive activation of Ras signaling is very common in the hepatocellular carcinoma (HCC). Therefore, we firstly examined whether the expression of CARF was regulated by the oncogenic Ras signaling. Over-expression of HA-Ras^V12^ (HA-tagged Ras^V12^) in L02 (normal hepatic cells) and 7404 (HCC cells) cells dramatically up-regulated the expression of CARF (Figure 1A). Next, we examined the expression of CARF in the liver tissues of the HCC mouse model (Alb-Cre; P53^f/f^; Loxp-Stop-Loxp-Ras^G12D^). It was found that the mRNA level and protein level of CARF were increased in the HCC mouse model compared with their control littermates (P53^f/f^; L-S-L-Ras^G12D^) (Figure 1B–1C). These observations suggested the up-regulation of CARF in the cell malignant transformation. To study the roles of CARF in the malignant transformation, the expression of CARF was knocked down in L02 cells during the transformation driven by HA-Ras^V12^. Down-regulating the expression of CARF significantly impaired the malignant transformation (Figure 1D). Taken together, these results indicated that the expression of CARF was induced by the oncogenic Ras and necessary for the malignant transformation.

### The expression of CARF was up-regulated in human HCC samples

**Figure 2:**
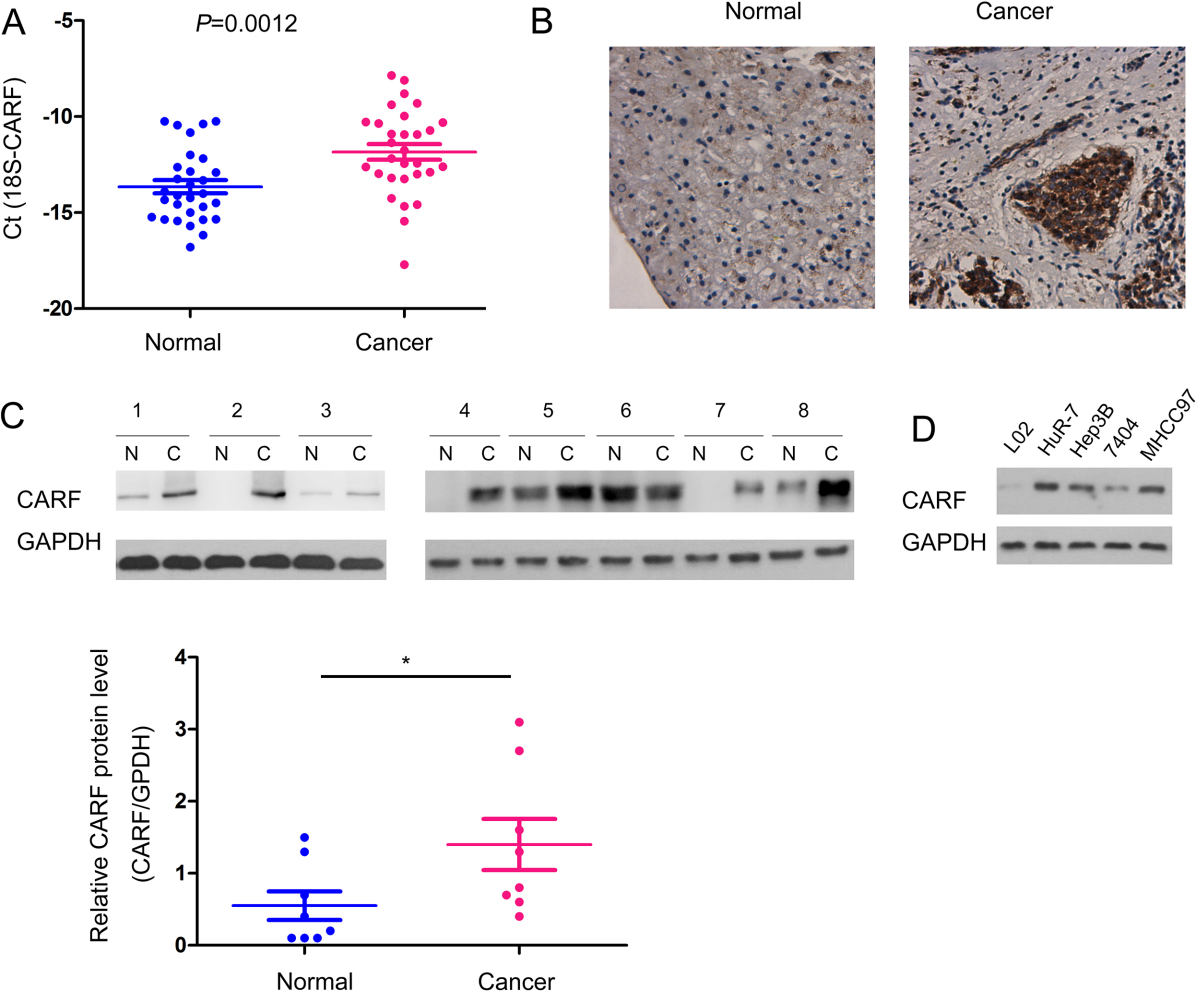
The expression of CARF was up-regulated in human HCC samples (**A**) Up-regulation of CARF mRNA in the human HCC clinical samples compared with the paired adjacent non-cancerous tissues. The mRNA level of CARF in 30 HCC clinical samples and paired non-cancerous tissues was examined using Real-time PCR. (**B–C**) Up-regulation of CARF protein level in the human HCC clinical samples compared with the paired adjacent non-cancerous tissues. The protein level of CARF in HCC clinical samples and paired non-cancerous tissues was examined using immunohistochemistry (B) and western blot (C). The protein level of CARF in (C) was quantified. (**D**) The expression of CARF in normal hepatic cells (L02) and HCC cells (HuR-7, Hep3B, 7404, MHCC97).

We next extended our study to the human HCC samples. The mRNA level of CARF in 30 HCC tissues and paired adjacent tissues was examined using Real-time PCR. As shown in Figure 2A, CARF mRNA level was increased in human HCC samples (Figure 2A). Moreover, the protein level of CARF was elevated in the cancerous tissues, which was demonstrated by the immunohistochemistry and Western blot analysis (Figure 2B–2C). In addition, we examined the expression of CARF in a panel of HCC cell lines and normal L02 cells. The lowest expression of CARF was found in L02 cells (Figure 2D). Collectively, these results demonstrated that the expression of CARF was up-regulated in HCC tissues and cell lines.

### CARF promoted the growth and migration of HCC cells

**Figure 3:**
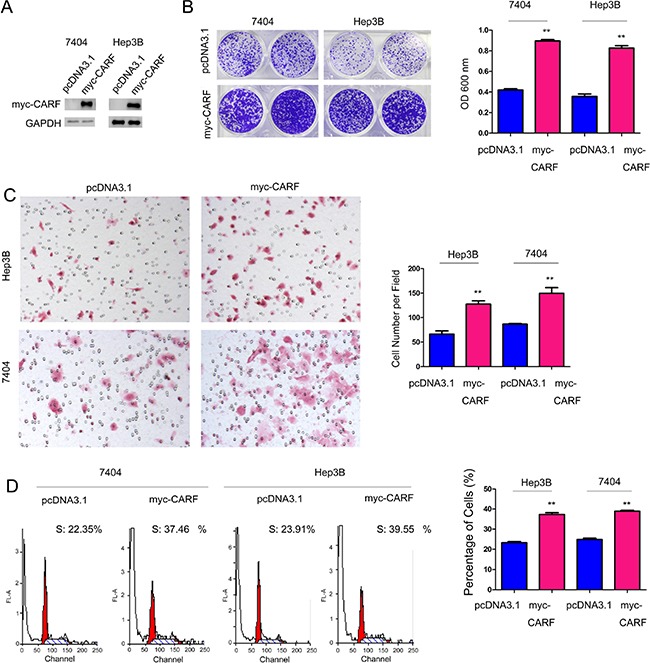
CARF promoted the growth and migration of HCC cells (**A**) Forced expression of myc-tagged CARF in 7404 and Hep3B cells. (**B**) Over-expression of CARF promoted the growth of 7404 and Hep3B cells demonstrated by the crystal violet assay. The experiments were performed in triplicates. (**C**) Over-expression of CARF promoted the migration of 7404 and Hep3B cells demonstrated by the Boyden Chamber assay. The experiments were performed in triplicates. (**D**) Over-expression of CARF altered the distribution of the cell cycle and increased the percentage of cells in S phase. The percentage of cells in S phase was statistically analyzed. The experiments were performed in triplicates. **P < 0.01.

To study the biological functions of CARF in the progression of HCC, we firstly forced the expression of CARF in 7404 and Hep3B cells (Figure 3A). Up-regulation of CARF in 7404 and Hep3B cells promoted cell growth in the crystal violet assay (Figure 3B). Also, forced expression of CARF promoted the migration of 7404 and Hep3B cells in the Boyden chamber assay (Figure 3C). Consistent with the observations in the crystal violet assay, the expression of CARF altered the distribution of the cell cycle with more cells in the S phase, suggesting that CARF could promote the proliferation of HCC cells (Figure 3D).

**Figure 4:**
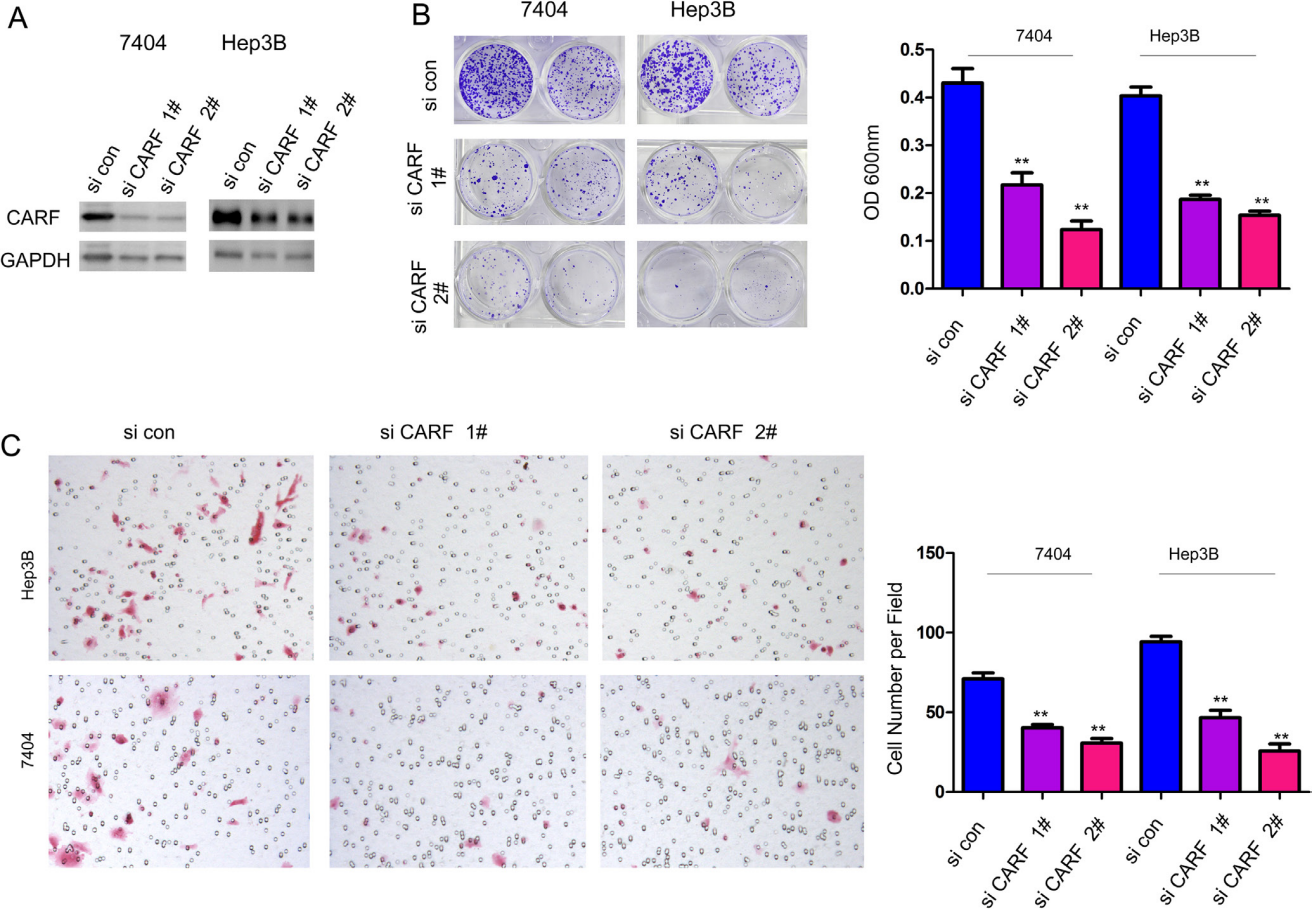
Knocking down the expression of CARF inhibited the growth and migration of HCC cells (**A**) Knocking down the expression of CARF in 7404 and Hep3B cells. (**B**) Down-regulation of CARF inhibited the growth of 7404 and Hep3B cells demonstrated by the crystal violet assay. The experiments were performed in triplicates. (**C**) Down-regulation of CARF inhibited the migration of 7404 and Hep3B cells demonstrated by the Boyden Chamber assay. The experiments were performed in triplicates. **P < 0.01.

In the next study, we investigated the roles of endogenously expressed CARF. At least 50% CARF expression was knocked down by two independent siRNA sequences (Figure 4A). Down-regulating the expression of CARF inhibited the growth and migration of 7404 and Hep3B cells in the crystal violet assay and Boyden chamber assay, respectively (Figure 4B–4C). Collectively, these results showed that CARF played a positive role in the progression of HCC.

### CARF activated beta-catenin/TCF signaling and interacted with beta-catenin

**Figure 5:**
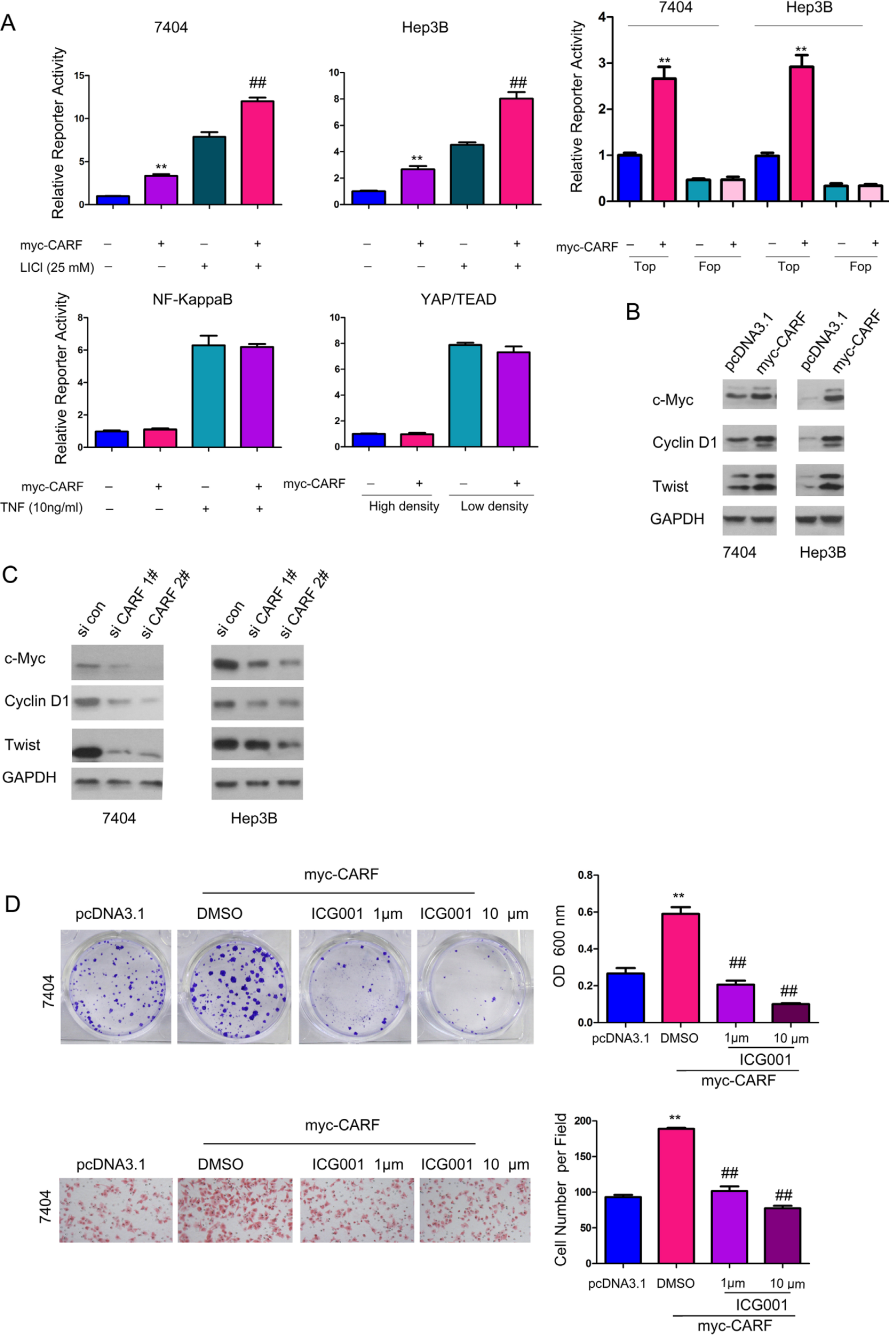
CARF activated beta-catenin/TCF signaling in HCC cells (**A**) Over-expression of myc-tagged CARF (myc-CARF) activated the Topflash reporter in 7404 and Hep3B cells, while the expression of CARF did not affect the activity of NF-kappaB and YAP/TEAD signaling. The experiments were performed in triplicates. Fopflash (Fop) was used as a negative control. (**B**) Over-expression of myc-tagged CARF (myc-CARF) activated the expression of target genes downstream of beta-catenin/TCF signling in 7404 and Hep3B cells. (**C**) Knocking down the expression of CARF inhibited the expression of target genes downstream of beta-catenin/TCF signling in 7404 and Hep3B cells. (**D**) The inhibitor of beta-catenin/TCF signaling, ICG001, abolished the promoting effects of CARF on the growth and migration of 7404 cells in a dose-dependent manner. The experiments were performed in triplicates. **P < 0.01; ^##^*P* < 0.01.

To find out the molecular mechanism through which CARF promoted the growth and migration of HCC cells, we performed the screening using reporter assay targeting various signaling pathways. In the screening, CARF was found to activate Topflash reporter (a reporter for beta-catenin/TCF signaling) while exerted few effects on the activation NF-kappB and YAP/TEAD signaling (Figure 5A). In the resting state, overexpression of myc-CARF elevated the activity of Topflash to three-four folds compared with the control group (Figure 5A). However, overexpression of myc-CARF did not affect the activity of Fopflash, the negative control of Topflash (Figure 5A). Moreover, CARF potentiated the activation of Topflash reporter by LiCl (Figure 5A). In addition, overexpression of CARF in 7404 and Hep3B cells up-regulated the expression of several target genes downstream of beta-catenin/TCF signaling, such as Cyclin D1, Twist and c-Myc (Figure 5B), while down-regulation of CARF inhibited the expression of these target genes (Figure 5C). These results suggested that CARF activated beta-catenin/TCF signaling in HCC cells. In addition, ICG001, the inhibitor for beta-catenin/TCF complex, abolished the promoting effects of CARF on the growth and migration of 7404 cells (Figure 5D), suggesting that CARF promoted the progression of HCC by positively regulating beta-catenin/TCF signaling.

**Figure 6:**
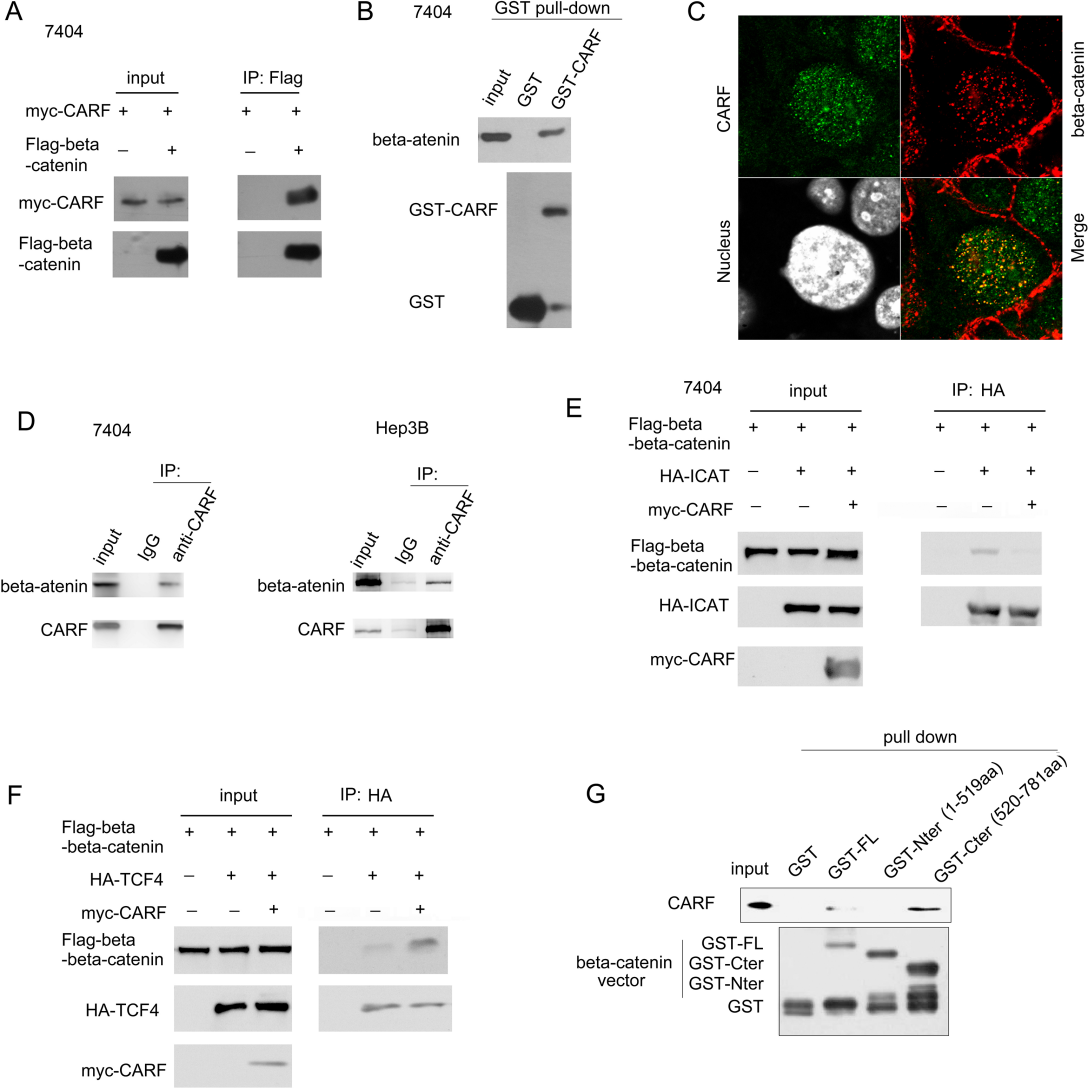
CARF interacted with beta-catenin in HCC cells (**A**) Exogenously expressed beta-catenin (Flag-beta-catenin) and CARF (myc-CARF) interacted with each other in the immunoprecipitation assay in 7404 cells. (**B**) GST-fused CARF (GST-CARF) binded the endogenously expressed beta-catenin. The GST-CARF fusion protein was purified and incubated with the 7404 cell lysate. Details about GST pull-down assay were described in the “Materials and Methods”. (**C**) Co-localization of CARF and beta-catenin was examined using fluorescence. The green signals were for CARF, and the red signals were for beta-catenin. (**D**) Endogenously expressed beta-catenin and CARF interacted with each other in the immunoprecipitation assay in 7404 and Hep3B cells. (**E**) Over-expression of CARF inhibited the interaction between beta-catenin and ICAT. (**F**) Over-expression of CARF enhanced the interaction between beta-catenin and TCF4. All of the experiments were performed three times. (**G**) GST pull-down assay was used to identify the domains mediating the interaction between beta-catenin and CARF. The fusion protein of GST-full length beta-catenin (GST-FL), GST-C terminal of beta-catenin (GST-Cter) and GST-N terminal of beta-catenin (GST-Nter) was purified and the pull down assay was performed using 7404 cell lysate.

In the next study, we investigated how CARF regulated beta-catenin/TCF signaling. We first examined the interaction between CARF and beta-catenin. As shown in Figure 6A, exogenously expressed CARF (myc-CARF) and beta-catenin (Flag-beta-catenin) formed a complex (Figure 6A). In the GST pull-down assay, the fusion protein GST-CARF was found to bind beta-catenin (Figure 6B). Moreover, the endogenously expressed CARF and beta-catenin was found to co-localize and interact with each other (Figure 6C–6D). ICAT has been reported to inhibit the interaction between beta-catenin and TCF and impaired the activation of beta-catenin/TCF signaling. Therefore, we next examined whether CARF affected the interaction between ICAT and beta-catenin. It was found that over-expression of CARF attenuated the interaction between beta-catenin and ICAT (Figure 6E) and enhanced the interaction between TCF and beta-catenin (Figure 6F). Next, we determined the domain in the beta-catenin protein to interact with CARF. It was found that the C terminal of beta-catenin mediated its interaction with CARF (Figure 6G). In summary, CARF promoted the progression of HCC by positively regulating beta-catenin/TCF signaling.

### Knocking down the expression of CARF impaired the tumorigenesis of HCC in the Alb-Cre; P53^f/f^; Ras mouse model

**Figure 7:**
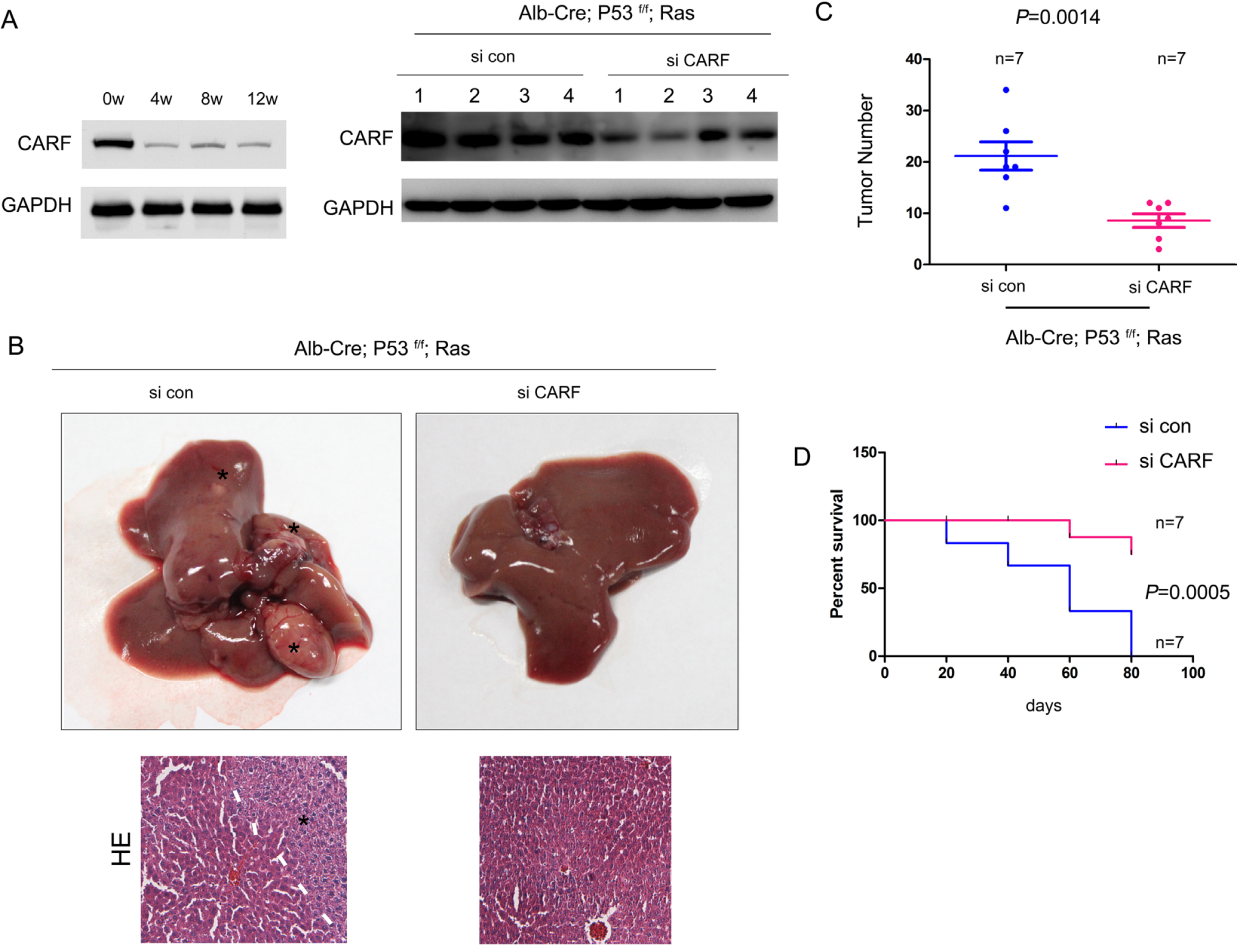
Knocking down the expression of CARF inhibited the tumorigenesis in the HCC mouse model (Alb-Cre; P53^f/f^; Loxp-Stop-Loxp-Ras^G12D^) (**A**) Adeno-associated virus-mediated knocking down the expression of CARF in the liver of HCC mouse model six weeks after adenovirus injection. In the left, the knocking down efficiency was examined at the indicated point. (**B**) The morphology of the tumors six weeks after adenovirus injection. The HE staining of the liver was shown below. The asterisk indicated the tumor region. (**C**) Knocking down the expression of CARF inhibited the tumorigenesis in the HCC mouse model demonstrated by the tumor number. (**D**) Knocking down the expression of CARF improved the survival of HCC mouse model.

We next examined the biological functions of CARF by turning to the HCC mouse model (Alb-Cre; P53^f/f^; Ras). About 50% CARF expression was knocked down using adeno-associated virus (AAV) (Figure 7A). The knocking down effects of CARF could be detected 12 weeks after the virus treatment. Knocking down the expression of CARF inhibited the tumorigenesis driven by the P53 loss and oncogenic Ras (Ras^G12D^) six weeks after adenovirus injection, which was demonstrated by the liver morphology and tumor number (Figure 7B– 7C). Moreover, down-regulation of CARF improved the survival of the HCC mouse model (Figure 7D). Taken together, these data suggested that CARF promoted the progression of HCC *in vivo*.

## DISCUSSION

The inactive mutation of P53 and constitutive activation of Ras signaling are very common in the carcinogensis of hepatocellular carcinoma (HCC) [13]. Restoration the function of P53 and inhibiting the oncogenic activity of Ras are the common strategies for HCC therapy. In this study, we have identified that CARF was a downstream target gene of oncogenic Ras signaling and necessary for the malignant transformation driven by Ras^V12^, suggesting that targeting CARF might be a promising strategy for blocking onogenic Ras signaling. Furthermore, previous reports have shown that CARF promoted carcinogenesis in cells losing wild type P53 function [10]. Due to inactivation of P53 in most of HCC samples, it is probable that the therapeutic strategy targeting CARF would show little effects on the normal hepatic cells. Therefore, CARF seemed to be a promising therapeutic target for HCC driven by P53 inactivation and Ras active mutation.

Previous studies demonstrated that the function of CARF depended on the status of P53. CARF induced cell growth arrest in cells with wild type P53, whereas it induced carcinogenesis in cells with absence of P53. In this study, it has been observed that the expression of CARF was up-regulated [10]. Combining with the frequent inactive mutation of P53 in HCC, it was reasonable that CARF would show oncogenic activity in HCC. Consistent with this hypothesis, the expression of CARF in HCC cell lines and mouse models promoted cell growth, migration and tumorigenesis.

In this study, we have shown that CARF, a Ras-induced gene, positively regulating beta-catenin/TCF signaling. These observations suggested that CARF might be a knot which mediated the cross-talk between Ras signaling and beta-catenin/TCF signaling. In fact, a few reports have shown the cross-talk between Ras signaling and beta-catenin/TCF signaling in HCC. For example, Sorafenib, the inhibitor of Ras signaling, inhibited Wnt/beta-catenin signaling and sensitized cells to the cisplatin treatment, suggesting the regulation of Wnt/beta-catenin signaling by Ras signaling [9]. However, the molecular mechanism remained unknown. The results presented in this study indicated that the expression of CARF was one of the mechanisms linking Wnt/beta-catenin signaling and Ras signaling.

Numerous studies have demonstrated that beta-catenin/TCF signaling is activated in HCC. Genetic disruption of APC leads to the activation of beta-catenin/TCF signaling and tumorigenesis of HCC [14]. However, the development of small molecular inhibitors targeting beta-catenin/TCF signaling is very difficult for its important roles in the physiological conditions. CARF, expressing in the cancer cells with inactivation of P53, might be a promising target. Additionally, CARF and ICAT have been demonstrated to compete for binding beta-catenin in this study. Up-regulation of CARF would disrupt this balance and lead to the activation of beta-catenin/TCF signaling.

In summary, the present study has shown the oncogenic roles of CARF in HCC by positively regulating beta-catenin/TCF signaling and suggesting that CARF might be a target for HCC treatment. Moreover, based on the expression pattern of CARF in HCC samples, it is very important to evaluate whether CARF might be served as tumor marker using HCC tissue array.

## MATERIALS AND METHODS

### Cell culture

The normal hepatic cells L02 and HCC cells (HuR- 7, Hep3B, 7404 and MHCC97) were purchased from ATCC (American Typical Culture Center) and cultured in DMEM medium containing 10% FBS (Fetal Bovine Serum, Sigma) and antibiotics (Sigma). Cells were incubated in the 37°C atmosphere containing 5% CO_2_.

### Mouse model

HCC mouse models (Alb-Cre; P53^f/f^; Loxp-Stop-Loxp-Ras^G12D^) were generated by crossing Alb-Cre mice with P53^f/f^; Loxp-Stop-Loxp-Ras^G12D^ mice. The expression of Alb-Cre led to the inactivation of P53, deleted the stop codon in the front of Ras locus and activated the expression of oncogenic Ras. Alb-Cre mice and P53^f/f^; Loxp-Stop-Loxp-Ras^G12D^ mice were bought from Jackson Lab (US, Canada & Puerto Rico). Littermates without the expression of Alb-Cre were used as control. All mouse experiments were performed under the approval of the Jiangsu University IACUC.

### Clinical samples

A total of 30 HCC tissues and paired non-cancerous tissues were obtained from patients who received surgery at the Affiliated Hospital of Jiangsu University. The written informed consent was obtained from the patients. This study was approved by the ethics committee of the hospital. All of the patients agreed with this study. Tissues and paired non-cancerous tissues were stored at −80°C in a freezer.

### Plasmid construction and transfection

The coding sequence of CARF was amplified and inserted into the expression vector pcDNA3.1 (CloneTech) and fused with the myc tag. The pcDNA3.1 plasmid and the CARF expression vector were transfected into HCC cells using lipofectamin 2000 (Invitroen). The transfected cells were selected with G418 (Gibco) for two weeks. The resist cells were pooled and examined the expression of myc tagged CARF.

### Real-time PCR analysis

The complementary DNA (cDNA) was prepared using the kit purchased from Promega after the total RNA was extracted using Trizol (Invitrogen). The mRNA level of CARF in the HCC tissues and paired non-cancerous tissues was examined by quantitative real-time PCR using SYBR Green Realtime PCR Master Mix (TOYOBO) following the instructions of the manufacturer. Sequences of quantitative real-time PCR primers are listed as follows:

18S Forward primer: 5′-TAAATCAGTTATGGTT CCTT -3′

18S Reverse primer: 5′-CGACTACCATCGAAAG TTGA-3′

CARF Forward primer: 5′-TCATCTCCTTTTCCAT GGCC-3′

CARF Reverse primer: 5′-TCTTGGCAACCAGTTC ATCT-3′

### Crystal violet assay

1 × 10^3^ cells were seeded in 12-well plates. Medium was changed every two days. Two weeks later, the cells were stained with 0.5% crystal violet solution. After incubation for 5 min, the cells were washed twice with phosphate-buffered saline (PBS) and photographed. Cells were resolved with 1% SDS solution. The absorbance at 600 nm was measured using a microplate reader.

### Boyden chamber assay

Boyden chamber (Neuro probe) was used to evaluate the migratory ability of HCC cells. Cells (2 × 10^5^) was suspended in 50 μl medium containing 1% FBS and placed in the upper chamber. The lower chamber was loaded with 152 μl medium containing 10% FBS. 8 hours later, cells migrated to the lower surface of filters was detected with traditional hematoxylin and eosin (H&E) staining. The experiments were repeated for thrice. Four random visual fields were counted for each sample and the average was determined.

### Cell cycle analysis

Cells were fixed with 70% ethanol, stained with PI (Propidium Iodide), and digested with RNase. Then, the cell cycle distribution was analyzed with flow cytometry (BD Bioscience).

### Western blotting

Cells were lysed in lysis buffer (Cell Signaling Technology). After being determined the protein concentration, the cellular protein was subjected to SDS-PAGE and western blot analysis with the following primary antibodies. HA (1:5000) and Flag HA (1:5000) tag antibodies were obtained from Sigma, CARF antibody was bought from Abcam HA (1:3000), GAPDH antibody was bought from Santa Cruz (1:3000), c-Myc, CyclinD1, Twist and beta-catenin (1:1000) antibodies were obtained from Cell Signaling Technology.

### Knocking down the expression of CARF in HCC cells and *in vivo*

The lentivirus and adeno-associated virus (AAV) for knocking down the expression of CARF were purchased from Genechem (Shanghai, China). For down-regulating the expression of CARF in HCC cells, si CARF lentivirus and control lentivirus were incubated with 7404 and Hep3B cells overnight, then cells were sorted by GFP. For down-regulating the expression of CARF in HCC mouse model, 2 × 10^9^ PFU si CARF adeno-associated virus (AAV) and control virus were injected into the tail vein. The down-regulation of CARF was examined.

### Immunohistochemistry

5 μm-thick consecutive sections of HCC tumor tissues and paired normal tissues were cut and mounted on glass slides. After deparaffinizing, rehydrateing, antigen retrieval and blocking endogenous peroxidases, the sections were washed thrice with 0.01 mol/l PBS (8 mmol/l Na_2_HPO_4_, 2 mmol/l NaH_2_PO_4_ and 150 mmol/l NaCl) for 5 min each, blocked for 1 h in 0.01 mol/l PBS supplemented with 0.3% Triton X-100 and 5% normal goat serum, followed by addition of anti-CARF (1:100, Abcam) antibody at 4°C overnight. After brief washes in 0.01 mol/l PBS, sections were exposed for 2 h to 0.01 mol/l PBS containing horseradish peroxidase-conjugated rabbit anti-goat IgG (1:500), followed by development with 0.3% H_2_O_2_ and 0.03% 3,30-diaminobenzidine in 0.05 mol/l Tris-HCl (pH 7.6). Immunohistochemistry for each sample was performed at least three separate times, and all sections were counterstained with hematoxylin.

### Immunoprecipitation

Cells were washed with ice-cold PBS and harvested in Tris-buffered saline (pH 7.4), containing 50 mM Tris, 150 mM NaCl, 1% NP-40, 1 mM EDTA, 1 mM Na_3_VO_4_, 10 mM NaF, 2.5 mg/ml aprotinin and leupeptin, 1 mM beta-glycerophosphate and AEBSF (4-(2-aminoethyl) benzenesulfonyl fluoride hydrochloride) and 10 mM iodoacetate. Cell lysate was incubated on ice for 15 min before cellular debris and nuclei were removed by centrifugation at 10000 g for 15 min. Cell lysate was incubated with the antibody overnight at 4°C. Protein A-Sepharose (Amersham Biosciences, Piscataway, NJ, USA) beads in a 50:50 mixture in 50 mM Tris buffer (pH 7.0) were added to the cell lysate and further incubated for another 4 h at 4°C. The immunoprecipitates were washed four times with Tris-buffered saline and boiled for 5min in 40 μl Laemmli buffer containing 0.02% blue bromophenol and 2% bmercaptoethanol.

### Statistical analysis

Statistical analyses were performed by the Student *t-test* (two-tailed) using Prism GraphPad software. Differences with *P* < 0.05 were considered statistically significant. Data were represented as mean ± SEM.
